# *In vivo* blockade of 5HT3 receptors in the infralimbic medial prefrontal cortex enhances fear extinction in a rat model of PTSD

**DOI:** 10.22038/ijbms.2021.54299.12197

**Published:** 2021-06

**Authors:** Ahmad Mohammadi-Farani, Mahdi Taghadosi, Sara Raziee, Zahra Samimi

**Affiliations:** 1 Pharmaceutical Sciences Research Centre, Health Institute, Kermanshah University of Medical Sciences, Kermanshah, Iran; 2 Department of Pharmacology and Toxicology, School of Pharmacy, Kermanshah University of Medical Sciences, Kermanshah, Iran; 3 Department of Immunology, School of Medicine, Kermanshah University of Medical Sciences, Kermanshah, Iran; 4 Student Research Committee, Kermanshah University of Medical Sciences, Kermanshah, Iran

**Keywords:** Fear extinction, Infralimbic medial - prefrontal cortex, PTSD, Single prolonged stress, 5HT3 receptor

## Abstract

**Objective(s):**

Treatments that reverse deficits in fear extinction are promising for the management of post-traumatic stress disorder (PTSD). 5-Hydroxytryptamine type 3 (5-HT3) receptor is involved involved in the extinction of fear memories. The present work aims to investigate the role of 5HT3 receptors in the infralimbic part of the medial prefrontal cortex (IL-mPFC) in extinction of conditioned fear in the single prolonged stress (SPS) model of PTSD in rats.

**Materials and Methods:**

The effect of SPS administration was evaluated on the freezing behavior in contextual and cued fear conditioning models. After the behavioral tests, levels of 5HT3 transcription in IL-mPFC were also measured in the same animals using the real-time RT-PCR method. To evaluate the possible role of local 5HT3 receptors on fear extinction, conditioned freezing was evaluated in another cohort of animals that received local microinjections of ondansetron (a 5HT3 antagonist) and ondansetron plus a 5HT3 agonist (SR 57227A) after extinction sessions.

**Results:**

Our findings showed that exposure to SPS increased the freezing response in both contextual and cued fear models. We also found that SPS is associated with increased expression of 5HT3 receptors in the IL-mPFC region. Ondansetron enhanced the fear of extinction in these animals and the enhancement was blocked by the 5HT3 agonist, SR 57227A.

**Conclusion:**

It seems that up-regulation of 5HT3 receptors in IL-mPFC is an important factor in the neurobiology of PTSD and blockade of these receptors could be considered a potential treatment for this condition.

## Introduction

Post-traumatic stress disorder (PTSD) is a psychiatric condition that is believed to be related to impaired control of fear emotions (1). Fear conditioning is a kind of associative learning paradigm that is widely used for studying fear-related responses in animals (2). In this model, subjects learn to associate a conditioned stimulus (CS), such as a context (training chamber) or a tone (cue), with an unconditioned fear-inducing stimulus (US), such as an electrical foot shock. Later if the animals are exposed to CS alone, they show fear responses that are normally elicited by US. Repeated re-exposure to CS without US gradually alleviates the fear response. This phenomenon is called “fear extinction” (3). Extinction memory is a newly formed memory that competes with the previous fearful memory. Its formation requires complex neural circuits mainly involving three brain regions; the medial prefrontal cortex (mPFC), hippocampus (HPC), and amygdala (4). It is strongly believed that deficits occurring in the extinction process are the underlying cause of PTSD. Therefore, any drug that is capable of weakening the traumatic memories or reinforcing the extinction of conditioned fear in animal models would be promising in the treatment of PTSD (5-9). 

The role of 5-hydroxytryptamine (5HT) and the serotonergic system is largely investigated in conditioning and extinction processes. Different 5HT receptors such as 5HT1A (10-12), 5HT2A (13), 5HT2C (14), 5HT3 (15-17), and newly described serotonin receptors like 5-HT6, 5-HT7, and 5-HT5A (18) are found to play a role.

The 5-HT3 receptor is an ionotropic receptor consisting of two subunits, 5-HT3A and 5-HT3B. The 5-HT3A subunit is essential to receptor function (19). Radio-ligand binding and *in situ *hybridization techniques have confirmed the presence of 5HT3A receptors in brain areas involved in the extinction process (20, 21). Previous research shows that central 5HT3 receptors are involved in the formation of mental conditions like depression (22, 23), anxiety (24, 25), memory disturbances (26-29), and social-behavioral disorders (30). Recently it is shown that 5HT3 receptors contribute to brain processes involved in the extinction of fearful memories (16). Accordingly, data from 5HT3 knockout mice revealed that presence of the 5-HT3A receptor is necessary for the extinction of contextual and cued fear (15). The exact sites and mechanism of action of these receptors in fear extinction are not determined yet.

The infra-limbic medial prefrontal cortex (IL-mPFC) is a cortical region that has a central role in the suppression of fear in rodents (31). Like other parts of mPFC, it contains high levels of 5HT3 receptors (32). As far as we know, there is no previous research on the role of 5HT3 receptors in IL-mPFC on extinction modulation in animal models. Our main goal in this study is to evaluate the effect of infra-limbic administration of ondansetron (as a 5HT3 antagonist) on extinction learning in the single prolonged stress (SPS) model of PTSD. We will use contextual and cued fear conditioning models in our research. Further behavioral experiments like the elevated plus maze (EPM) and open field (OF) tests are also performed for better interpretation of the results.

There is evidence that altered transcription of 5HT3 receptors may increase the vulnerability of animals to anxiety disorders (33, 34). In humans, changes in the regulation of the 5HT3A subunit have been described in psychiatric conditions like bipolar disorders (35). Based on the above we hypothesized that changes in the regulation of 5-HT3 receptors in IL-mPFC may be involved in the modulation of fear symptoms in PTSD. Therefore, in our experiments, we also evaluated the possible differences in the expression ratio of the 5HT3A receptor in mPFC of normal vs SPS rats. 

## Materials and Methods

***Animals***

Male adult Wistar rats (180–250 g) were used in the experiments. Animals were housed 5 per cage and maintained on a 12 hr light/dark cycle at an almost fixed temperature (22 °C). They had free access to food and water. All experiments were done in accordance with the Institutional Guide for the Care and Use of Laboratory animals (Ethical code, IR.KUMS.REC.1397.048). 

***Drugs***

SR 57227A (5-HT3 agonist) and Ondansetron (5-HT3 antagonist) were obtained from sigma- Aldrich (Sigma-Aldrich, Taufkirchen, Germany). Drugs were dissolved and diluted in 0.9% saline solution. They were injected in a volume of 0.5 µl per side at a rate of 0.5 µl/min.

***Single prolonged stress procedure***

The SPS procedure was performed as described elsewhere (36). Briefly, rats were restrained in a plastic restrainer for 2 hr. Then they were immediately put into a water bath (circular; 60 cm diameter, 35 cm depth, and 25 °C) for a 20 min forced swim period. After the swim, they were towel-dried and put to rest for fifteen minutes. In the final step, rats were exposed to diethyl ether until loss of consciousness, and then they were returned to their home cages.

***Stereotaxic surgery***

Rats were anesthetized with a cocktail of ketamine and xylazine (30 mg/kg and 5 mg/kg, respectively) and then, they were fixed in a stereotaxic instrument (WPI, berlin, Germany). A stainless-steel guide cannula (10 mm long, 22 gauge) was implanted bilaterally in the rat skull so that the tip of the cannula was located 1 mm above the medial prefrontal cortex. The cannula was fixed on the skull with dental cement. mPFC was positioned (AP: **+**3 mm from bregma, ML: ±0.8 mm, DV: -4 mm) according to the atlas of Paxinos and Watson (37). Correct implantation of the cannula was confirmed by injection of 1 µl methylene blue (5% in saline) in the IL-mPFC of random samples.

***Conditioning apparatus***

We used a slightly modified Ugo Basile (model 46002) fear conditioning apparatus to study the contextual and cued fear in rats. The system was controlled by AnyMaze software (Version 2.1; Ugo Basile). The conditioning box (context A) was a bottomless Plexiglas container (26×26×30 cm) which was enclosed inside a bigger soundproof chamber. The chamber contained a stainless steel electrified grid floor to deliver the unconditioned stimulus (US), a flashlight to deliver the conditioned stimulus (CS), a noiseless fan to provide fresh air, and a dimmed LED bulb to sustain the basal lighting. A video was recorded by a camera above the apparatus. This video was replayed by a blind observer and the time spent in the freezing state was determined. Freezing was recorded and defined as absence of movement lasting more than 1 sec, and was calculated according to the following formula: Percent freezing = total time freezing × 100 / total time of training)

***Conditioning and extinction tests***

**Figure 1:**
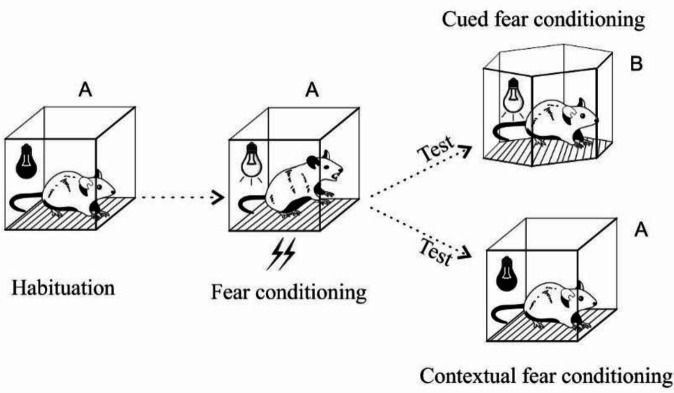
Fear conditioning tests included different phases of habituation (context A), fear conditioning training (context A), and contextual and cued fear conditioning tests (context A or B)

**Figure 2:**
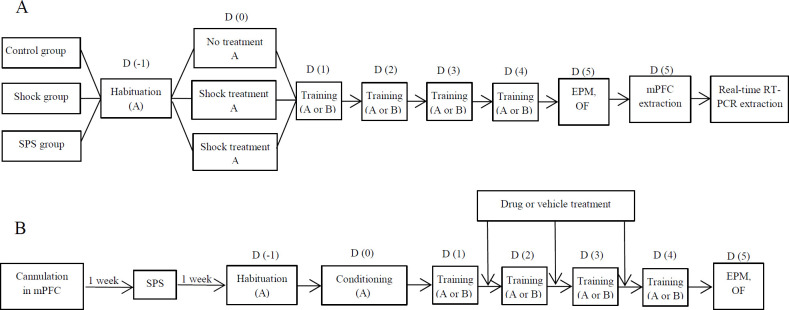
Timeline of behavioral and molecular experiments: a) experiments without drug treatment, b) experiments with drug treatment. A; Context A, B; Context b, EPM: Elevated plus maze test; D; day, OF: Open field test; SPS: Single prolonged stress

The conditioning and extinction procedures were performed according to Kumari *et al*. (38) with some modifications (Figure 1). Behavioral tests were started one week after SPS administration. On day -1, for habituation of the animals, rats were put in the apparatus in two sessions, 10 min each. On day 0, a conditioning session was started by putting the rats in context A of the apparatus. Here they received 7 trials of CS, 30 sec flashing house light (500 ms on/off, 8 lx), which was co-terminated with US, a foot-shock of 0.66 mA for 2 sec. The interval between each trial varied from 1 to 2 min. After 24 hr contextual fear extinction was tested by putting the animals back in the training box (context A) for a period of 5 min with no CS or US being applied. Freezing of rats was scored in this time interval. Cued fear extinction was tested in a different context (context B). It was cubical with a different size, wall texture, floor, and odor (mint extract). After 5 min of habituation, a 6.5 min test session started through which animals were presented with CS 5 times (no US was used in this session). Here, the total time of freezing was measured during each CS presentation. Isopropyl alcohol was used to clean the box before each experiment. A video was recorded in each test session and the freezing behavior was scored manually by a blind experimenter who watched the off-line films. The timeline for the acquisition and extinction experiments is represented in Figure 2.

***Experimental groups***

Two sets of experiments were conducted in this research. In the first set, the aim was to evaluate contextual and cued fear extinction in shocked, SPS, and control groups (Figure 2A). Three groups of rats were used in contextual and three in cued conditioning procedures (n=8 in each group). All groups were exposed to context A for habituation. The training tests were performed on four consecutive days. The following groups were used in the contextual paradigm: a) A control group that did not experience the SPS procedure and did not go through the shock step but underwent the training tests, b) A shock group that was similar to the control group except that they experienced the shock step 24 hr before the first extinction test, c) An SPS-group that underwent the SPS procedure one week before receiving the shock. The shock step was similar to the conditioning procedure described above. In the contextual paradigm, the training tests were held in context A of the conditioning apparatus. For the cued conditioning paradigm, three similar groups (n=8) underwent the same procedures, similar to the contextual design, except that the extinction tests were performed in a different context (context B).

The second set of experiments was meant to investigate the effect of a 5HT3 antagonist in the extinction of acquired fear (Figure 2B). Here again, three groups of rats were used for contextual and three for cued extinction tests. Habituation and conditioning were performed in context A for all groups. The control (vehicle) group in the contextual paradigm was conditioned 1 week after SPS administration. Twenty-four hours later, the first extinction training session was performed and it was repeated for 4 days. Immediately after each training, except on the fourth day, rats received microinjection (0.5 µl each side) of the vehicle. The other two groups went through the same procedures except that one group was injected, bilaterally, with ondansetron (2 µM/0.5 µl) and the other received R57227A (3 µM/0.5 µl) plus ondansetron (2 µM/0.5 µl) instead of the vehicle. Doses of the drugs were chosen based on our pilot study and previous studies using local injection of the drugs (17). SR57227A was administered 15 min prior to ondansetron. In the contextual paradigm context A was used for the training tests. The effect of drug treatment on cued fear extinction was evaluated in three other corresponding groups. The behavioral tests were similar to the contextual extinction groups except that the extinction tests were performed in context B of the conditioning apparatus.

***Elevated plus maze test***

The elevated plus-maze was a wooden structure made of two crossed open arms and two closed arms (50 × 12 cm) with closed arms having 35-cm-high black walls on both sides. The maze was established 50 cm above the ground. EPM was carried out the day after the last training session (Figure 2B). Experiments were performed 2 hr after rats were acclimatized to the room. The rats were put in the middle square of the maze facing an open arm. They were allowed to explore the maze for 10 min. After each test, the maze was cleaned with 70% ethanol. The behavior of the rat was recorded by a video tracking system (EthoVision XT8 video tracking system, Noldus, Netherlands). Behavioral measures were i) time spent in the open and closed arms ii) number of entries into the open and closed arms. Entry to an arm was defined as entering each arm with the four paws in that arm. Percentage of open arm time (OAT%) and open arm entry (OAE) were calculated as follows; OAT%= Time spent in the open arms × 100/ Time spent in all arms, OAE%= Entries into the open arm × 100/ Entries into all arms. 

***Open field test***

The open-field test was designed in accordance with the procedures described by Salehabadi *et al*. (39). One day after the last training trial, rats were placed in an open square (50*50*35) box. The floor was divided into 25 squares which were monitored by infrared beams. Square crossings, the number of crossings of animals in different squares, were counted as an index for locomotor activity. The test duration was 5 min.

***Real-time PCR***

One day after the last behavioral experiment, animals were decapitated, the brains were removed and the infralimbic part of the medial prefrontal cortices was isolated for PCR experiments. Total RNA was extracted from the frozen samples of IL-mPFC using the High Pure Tissue RNA Extraction Kit (Roche Applied Science, IN, USA) according to the manufacturer’s instructions. A PrimeScript RT reagent Kit (Takara, Shiga, Japan) was used to make cDNAs from the RNA. Amplification of cDNA was performed in a StepOnePlus real-time PCR System (Applied Biosystems, Foster City, CA, USA). Glyceraldehyde-3-phosphate dehydrogenase (GAPDH) was used as the internal standard. The primer sequences for 5HT3A and the internal standard are shown in Table 1. The total volume of the reaction was 25 µl which contained 1 µl of each primer, 12.5 µl of Power SYBR Green PCR Master Mix 2X (applied biosystems, CA, USA), 1.5 µl of the template, and 9 µl PCR grade water. The holding stage (95 °C for 15 min) was followed by the cycling stage (denaturation 30 sec in 95 °C, annealing 30 sec 60 °C, extention 30 sec in 72 °C) and the number of cycles was 45. Expression levels of 5HT-3A were compared between control and SPS rats using the 2^-ΔΔCt^ method (40). 

***Statistical analysis***

Data were analyzed using GraphPad Prism 8.0.2 (GraphPad Software, Inc., San Diego, CA, USA). Changes in the percentage of freezing in the extinction tests were compared by a two-way repeated measure (RM) ANOVA with day and group as the main factors. Results of EPM, OF, and PCR experiments were analyzed by one-way analysis of variance (ANOVA). Tukey *post hoc* analysis was performed to detect the significance of differences between groups. Data were expressed as mean ± SEM and *P*-values<0.05 were considered significant.

## Results

***Effects of SPS and shock on contextual and cued extinction***

**Figure 3:**
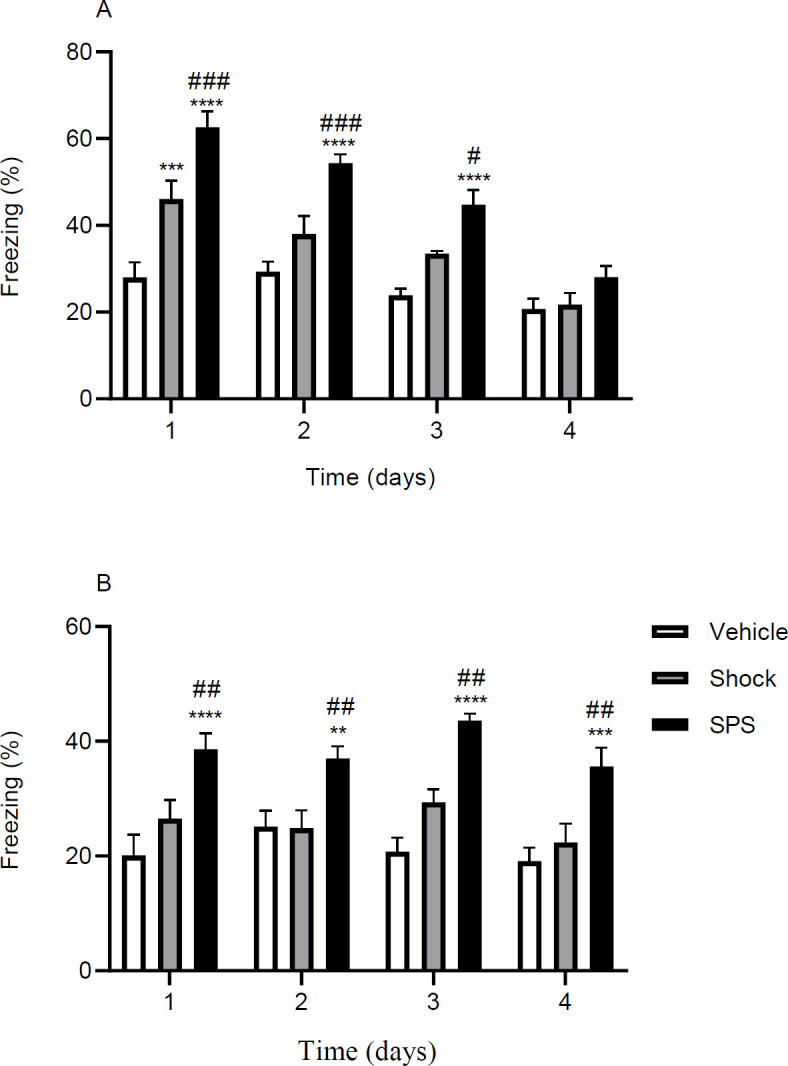
Role of SPS or shock on extinction learning in rats. Two cohorts of animals were used in these experiments and each cohort consisted of three groups. Animals in the contextual cohort (A) were pretreated in context A and trained in the same context for 4 consecutive days. The cued cohort (B) was put in context A for pretreatment procedures but was trained in another context (context B). Animals in the control group neither experience SPS nor received electrical foot shocks before the extinction training tests. The shock group received the conditioning foot-shocks before training but was not exposed to the SPS procedure. The SPS group went through the SPS phase one week before starting the experiments and received the shocks. Data are presented as Mean±SEM. ** *P*<0.01, *** *P*<0.001, **** *P*<0.0001 as compared with control group. # *P*<0.05, ## *P*<0.01, ### *P*<0.001 as compared with the shock group

To find the possible SPS-induced effects in the process of extinction learning, experiments were performed in contextual and cued fear extinction models in SPS, shocked, and vehicle-treated rats. Figure 3 shows the percentage of time spent freezing during the four days of extinction learning in these tests. Statistical analysis revealed that there was a significant difference in freezing time between different days and groups in contextual model [Two-way RM ANOVA: day; F (3, 63) = 35.4, *P*<0.0001, group; F (2, 21) = 43.03, *P*<0.0001, interaction; F (6, 63) = 4.34, *P*=0.001]. *Post hoc* tests showed that in the first three days of learning, the SPS group had an increased freezing response compared with the control group (*P*<0.0001). Comparison of the group means indicated that SPS rats tend to have the highest freezing time compared with other groups (Figure 3A).

In the cued extinction model, there was not a significant effect for the main factor of day [Two-way RM ANOVA: day; F (3, 63) = 2.05, *P*=0.11] but freezing differed between different groups [Two-way RM ANOVA: group; F (2, 21) = 39.49, *P*<0.0001]. Indeed, there was not a significant interaction between the two main factors [Two-way RM ANOVA: interaction; F (6, 63) = 0.80, *P*=0.56] (Figure 3B). Similar to the contextual model, animals in the SPS group had a higher freezing time compared with other groups. The results of the contextual and cued models indicate that regardless of the model of extinction, SPS rats have increased fear compared with the control group. 

***Effects of 5HT3 antagonist on contextual and cued extinction in SPS model***

**Figure 4:**
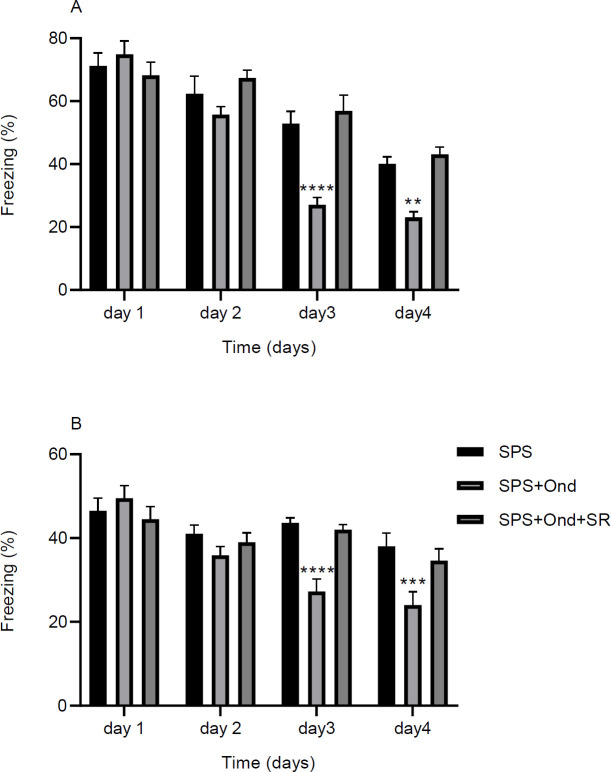
Role of 5HT3 receptor antagonist (ondansetron) in extinction learning in SPS rats. Two cohorts of animals were used in these experiments and each cohort consisted of three groups. All animals went through the SPS procedure one week before the start of the experiments. Habituation and conditioning took place in context A. Training was performed in context A for the contextual cohort (a) and context B for the cued cohort (b). The control group received intra-mPFC microinjections of the vehicle after each training session. The other two groups received either ondansetron or a 5HT3 agonist (SR 57227A) after the training sessions. Data are presented as Mean±SEM. ** *P*<0.01, *** *P*<0.001, **** *P*<0.0001 as compared with the SPS group

Earlier results showed that SPS rats have a deficient learning ability in the extinction learning tests (Figure 3). To evaluate the effects of 5HT3 receptors on the consolidation of extinction memory vehicle (0.5 µl), ondansetron (2 µM/0.5 µl) and ondansetron plus SR 57227A (3 µM/0.5 µl) were infused bilaterally into IL-mPFC of separate groups of SPS rats immediately after each extinction session. The tests were performed in the contextual and cued models (Figure 4). There was a significant effect for the main factors of day and treatment in the contextual [Two-way RM ANOVA, day: F (3, 63) = 88.52, *P*<0.0001. treatment: F (2, 21) = 8.53, *P*=0.001. interaction: F (6, 63) = 7.81, *P*<0.0001] and cued [Two-way RM ANOVA, day: F (3, 63) = 21.72, *P*<0.0001. treatment: F (2, 21) = 5.87, *P*=0.009. interaction: F (6, 63) = 5.12, *P*=0.0002] paradigms (Figure 4). In the contextual model ondansetron decreased freezing in the third and fourth days of the tests compared with the control group (*P*<0.0001, *P*<0.01, respectively). This effect was reversed by co-administration of SR 57227A (5HT3 antagonist) with ondansetron (Figure 4A). Results of the cued model were similar to the contextual model in that freezing was decreased in ondansetron treated rats in the third and fourth days of the experiments (*P*<0.0001, *P*<0.001, respectively), and co-treatment with SR 57227A blocked this effect (Figure 4B). 

***Effect of SPS and shock on the elevated plus-maze***

**Figure 5:**
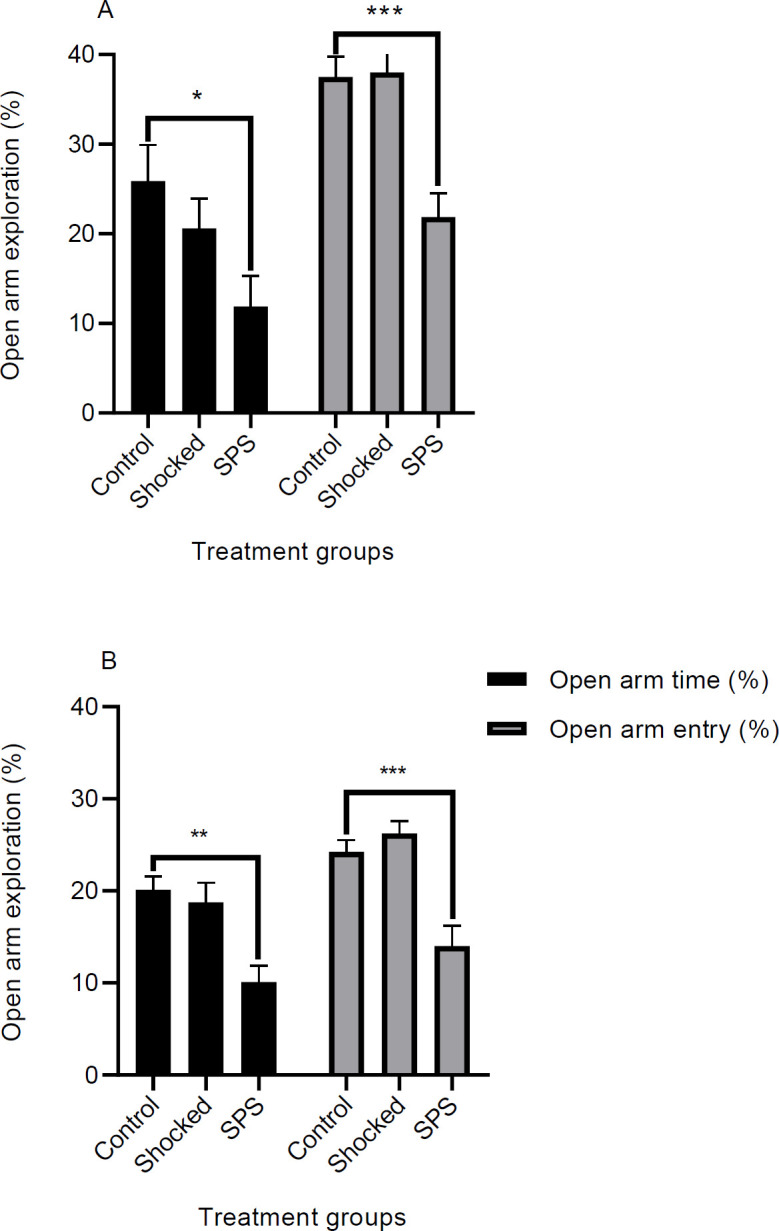
Effect of SPS or shock on anxiety level in the EPM test. Anxiety was tested one day after the last training session in two cohorts of rats. Each cohort consisted of three groups including a control group, a shock group, and an SPS group. The first cohort had experienced the contextual extinction tests (a) and the second cohort had gone under the cued extinction experiments (b) before the EPM tests. Time spent in the open arms and entries into the open arms were considered as indices for anxiety. Data are shown as Mean±SEM. * *P*<0.05, ** *P*<0.01, *** *P*<0.001 as compared with the control group. SPS: Single prolonged stress

The elevated plus-maze test was used to study the effects of shock and SPS on anxiety levels in the animals. The test was carried out one day after the last day of training in contextual and cued layouts (Figure 5). The percentage of time spent and the percentage of entries into the open arms were considered as common anxiety indices. A one way ANOVA revealed significant differences in “time spent in the open arms” and “entries into the open arms” in groups that underwent contextual [Time; F (2, 21) = 3.84, *P*=0.03, Entry; F (2, 21) = 14.17, *P*=0.0001] or cued [Time; F (2, 21) = 9.11, *P*=0.001, Entry; F (2, 21) = 15.6, *P*<0.0001] extinction training. As depicted in Figure 5, in both extinction models, SPS rats who were exposed to shock spent less time (contextual model; *P*<0.05, cued model; *P*<0.01) in the open arms of the maze and had fewer entries (contextual model; *P*<0.001, cued model; *P*<0.001) into the open arms. Although treatment of normal rats with shock tended to reduce the exploration time in the open arms, it was not statistically significant (*P*>0.05) compared with the control group (Figures 5A, B). Overall these results indicate that the shock-treated SPS group had increased levels of anxiety compared with normal rats and presentation of shock alone could not change the anxiety levels in the animals.

***Effect of SPS and shock on open field test***

**Figure 6:**
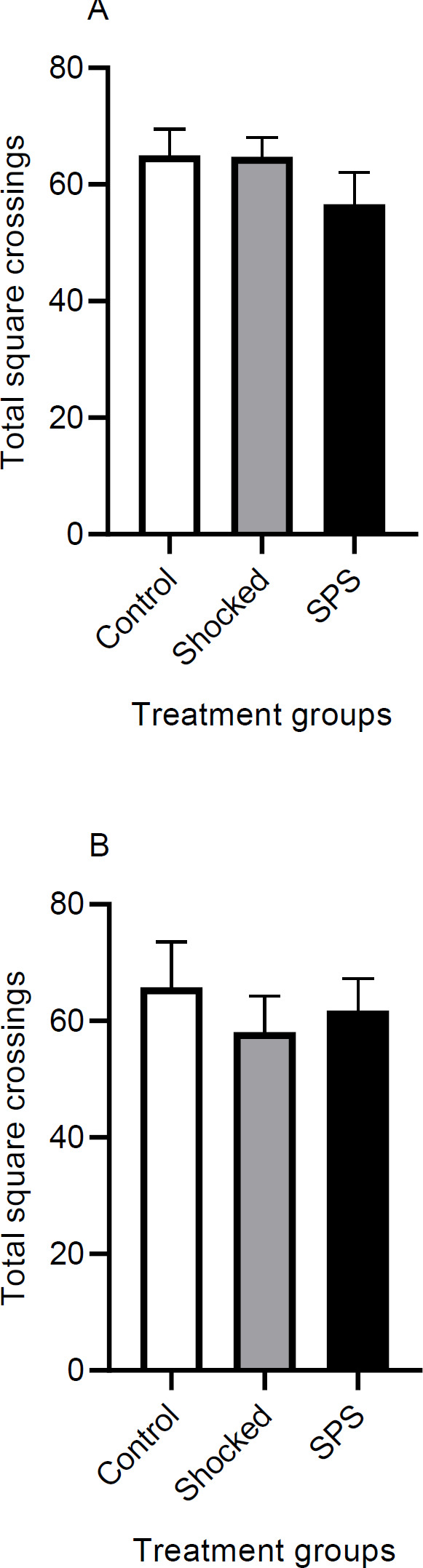
Effect of SPS and shock on locomotor activity in the open-field apparatus. The total square crossing was measured in the open field test in two cohorts of rats. The contextual cohort groups were tried on the contextual extinction tests before the open field experiments and consisted of a control, a shocked, and an SPS group (a). The cued cohort had the same corresponding groups and had experienced the cued extinction sessions (b). Data are displayed as Mean±SEM

To rule out the possible confounding effects of locomotion on anxiety indices in the EPM test, we evaluated the locomotor activity of non-drug-treated rats in the OF test one day after the last training session. The results show that there is not a significant difference between different groups in contextual [F (2, 21) = 1.13, *P*=0.33] (Figure 6A) or cued [F (2, 21) = 0.33, *P*=0.71] (Figure 6B) models. These results indicate that any change recorded in the EPM test is not due to motor function disturbances.

***Effects of 5HT3 antagonist on the elevated plus-maze in the SPS model***

**Figure 7:**
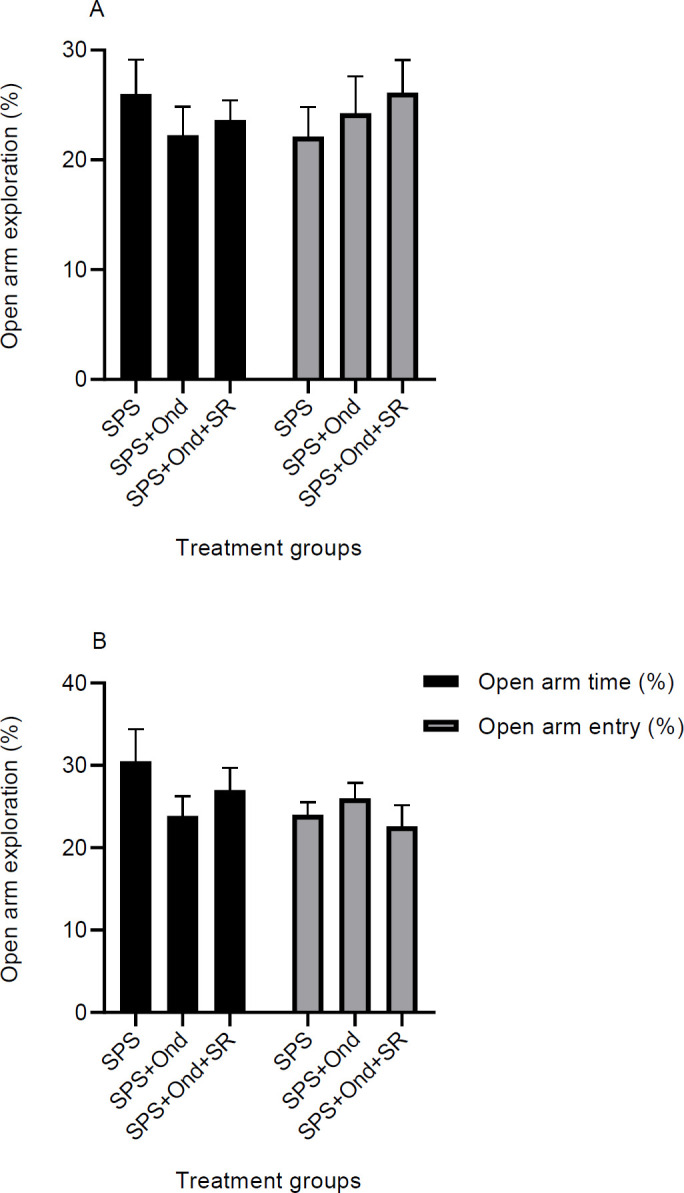
Effect of 5HT3 receptor antagonism on EPM test in SPS rats. Anxiety was tested one day after the last training session in contextual (a) and cued (b) cohorts of rats. Each cohort consisted of three groups. The first group was an SPS group that received intra-mPFC microinjections of the vehicle after each training session. The second group was injected with ondansetron (5HT3 antagonist), and the third group was treated with both ondansetron and SR 57227A (5HT3 agonist) after the training sessions. The contextual cohort had gone under contextual extinction tests and the cued cohort had experienced the cued extinction experiments before the EPM tests. Data are expressed as Mean±SEM

As shown in the previous results (Figure 5), SPS rats that were exposed to the shock experience of the conditioning phase, had increased anxiety compared with the normal rats. This part of the experiments was performed to find if ondansetron could change the anxiety levels in these rats. Animals treated with vehicle, ondansetron (2 µM/0.5 µl) or ondansetron plus SR57227A (3 µM/0.5 µl), were examined in the EPM test 24 hr after the last extinction training day (see Figure 2B). The EPM data indicated that there is no significant difference between groups in the time spent in open arms or arm entries for both contextual and cued models [Contextual, Time: F (2, 21) = 0.54, *P*=0.58, Entries: F (2, 21) = 0.43, *P*=0.65; Cued, Time: F (2, 21) = 1.17, *P*=0.32, Entries: F (2, 21) = 0.69, *P*=0.51] (Figures 7A, B). This simply means that antagonism of 5HT3 receptors in IL-mPFC is ineffective in relieving SPS rats from increased anxiety in the course of experiments. 

***Effects of 5HT3 antagonist on the open field test in the SPS model***

**Figure 8:**
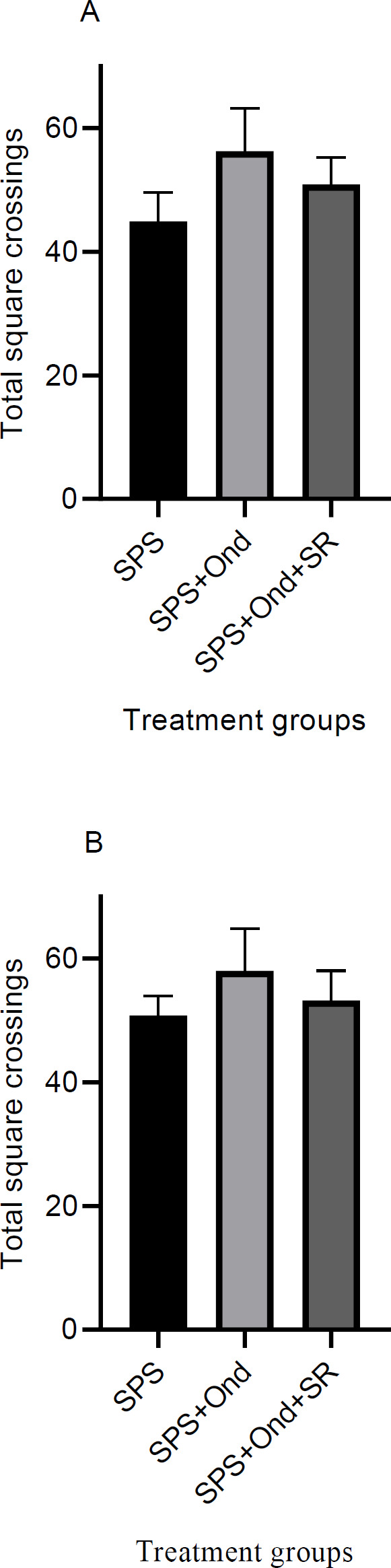
Effect of ondansetron on locomotor activity in the open field apparatus in SPS rats. Total square crossing was measured as the locomotor index. Two cohorts of animals were tested 24 hr after the last extinction test. The contextual cohort groups had previously passed the contextual extinction tests and consisted of SPS, ondansetron, and ondansetron plus SR 57227A (5HT3 agonist) groups (a). The cued cohort groups had the same corresponding groups and had experienced the cued extinction sessions (b). Data are displayed as Mean±SEM

Drug-treated SPS rats were evaluated for their motor function in the OF test one day after the extinction training experiments. Treatments [ondansetron (2 µM/0.5 µl) and ondansetron plus SR 57227A (3 µM/0.5 µl)] made no changes in the number of crossings between different groups that experienced contextual (Figure 8A) or cued (Figure 8B) extinction procedures [Contextual; F (2, 21) = 1.08, *P*=0.35, Cued; F (2, 21) = 0.23, *P*=0.79].

***Effects of SPS on 5HT3 receptor gene expression***

**Figure 9:**
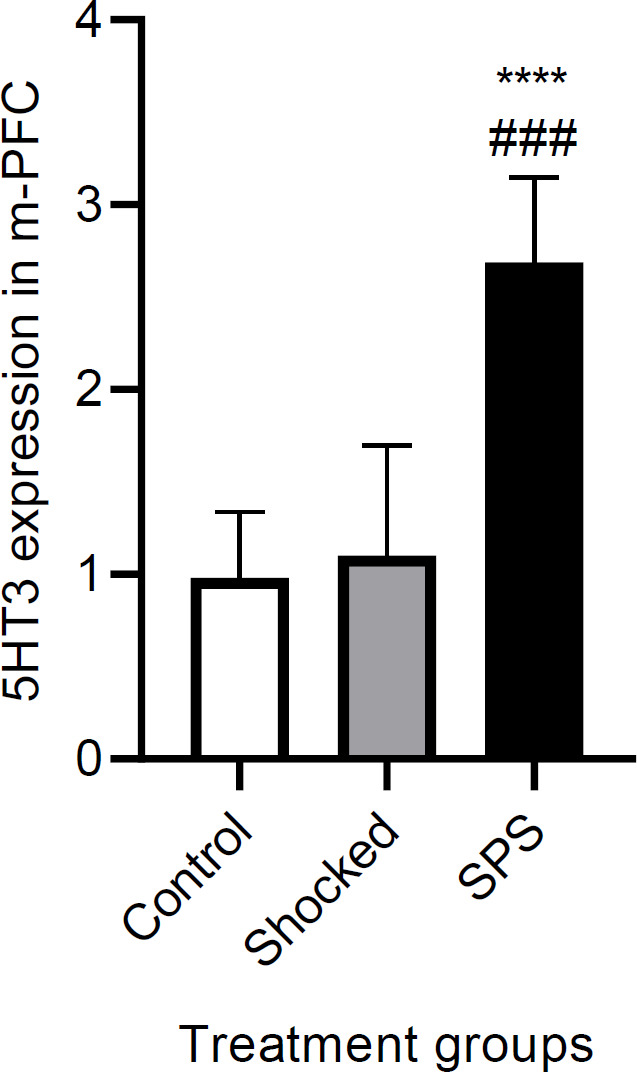
mRNA levels of 5HT3 receptors in the IL-mPFC . Data are presented as Mean±SEM. **** *P*<0.0001 as compared with the vehicle group. ### *P*<0.001 as compared with the shocked group

Comparison of the expression of 5HT3A receptors in IL-mPFC of shocked and SPS versus normal rats would give us a clue about the possible underlying changes in the neurobiology of SPS-induced PTSD and can further help to interpret the behavioral results from the drug-treated animals. Analysis of the data showed that mRNA expression differs between different treated groups [F (2, 15) = 23.63, *P*<0.0001 (n = 6)], with the SPS rats having increased expression compared with the vehicle (*P<*0.0001) or shocked (*P*=0.0001) groups (Figure 9). 

**Table 1:** Primer sequences used in RT-PCR

**Gene**		**Primer sequence**
**5HT3A**	**Forward**	**5´-ctc-ccc-tca-ttg-gtg-tct-ac-3´**
	**Reverse**	**5´-cgc-tgt-aaa-tcc-tgc-tta-tgc-3´**
**GAPDH**	**Forward**	**5´-tac-cag-ggc-tgc-ctt-ctc-ttg-3´**
	**Reverse**	**5´-gga-tct-cgc-tcc-tgg-aag-atg-3´**

SPS: Single prolonged stress

Ond: Ondansetron; SPS: Single prolonged stress; SR: SR 57227A

SPS: Single prolonged stress

Ond; Ondansetron, SPS: Single prolonged stress, SR: SR 57227A

Ond: Ondansetron, SPS: Single prolonged stress, SR: SR 57227A

SPS: Single prolonged stress

**Figure 10:**
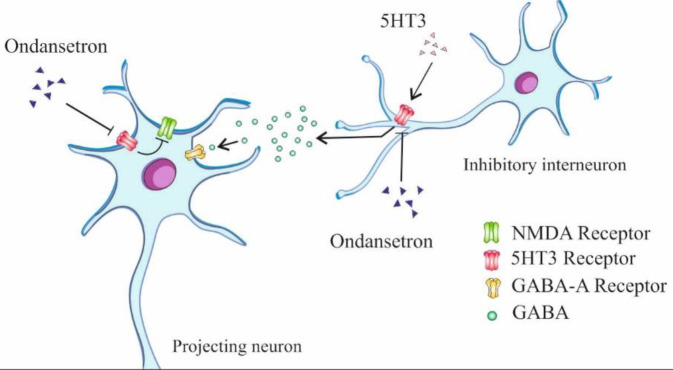
Schematic representation of the suggested roles of ondansetron on enhancing the inhibitory output neurons from mPFC. Either reducing GABA-A or increasing NMDA receptor activation in some projecting neurons in mPFC augments the excitatory potential of these neurons and ultimately results in suppression of fear presentation in conditioned models. Ondansetron decreases GABA release from the inhibitory interneurons by blocking presynaptic 5HT3 receptors on these cells; it also activates NMDA receptors on the projecting neurons through blockade of their inhibitory 5HT3 receptors

## Discussion

Our main results of this study is that (I) exposure to SPS, induces an impaired extinction of fear memory in contextual and cued fear conditioning models (II) 5HT3 receptors are up-regulated in the IL-mPFC of SPS rats, and (III) blocking of 5HT3 receptors in the IL-mPFC facilitates the extinction of contextual and cued fear memories without having effects on motor activity of the animals.

In the extinction learning experiments we found that in both contextual and cued conditioning models, the SPS rats had an overall higher freezing behavior. We also found that freezing in the cued model did not change over time (no significant effect for the main factor of day in the cued model), while it was reduced in the contextual model (significant effect for the main factor of day in the contextual model) (Figures 3A, B). This indicates that SPS animals are less inclined to lose sensitivity to the fear-related cues than the contextual features in which the conditioning has taken place. In other words, it implies that the cue, as an element in the environment, maybe a superior reminder of threat than the whole context in which the animals have experienced the traumatic event. It is actually in support of the notion that in SPS exposed rats elemental learning strategy (a learning strategy in which the association of different features of the environment is encoded individually with the aversive stimuli) predominates the conjunctive learning strategy (a learning strategy in which different elements in the environment are considered a whole with which the trauma is associated) (41). It is believed that elemental strategies mostly depend on the neocortex, while conjunctive strategies mostly depend on the hippocampus (42, 43).

Having found that SPS impairs extinction learning, we further found that microinjection of a 5HT3 blocker (ondansetron) into the IL-mPFC rescued fear extinction deficits in both cued and contextual models, and this effect was reversed by applying a 5HT3 agonist (SR 57227A) (Figure 4). The injections were made right after the training sessions. Therefore, we may conclude that 5HT3 receptors are probably involved in the consolidation phase of the extinction process. To rule out the possible effect of the shock itself, ondansetron and SR 57227A were also administered to a separate non-SPS group that received shocks, and the results indicated that there was not a significant difference for the main factors of day or shock exposure (the results are not shown). These data demonstrate that exposure to SPS is a predisposing factor for sensitivity of the animals to ondansetron.

In an early study, Yoshioka *et al*. (1995) showed that exposure of rats to an environment in which they had previously received foot-shocks raised the extracellular 5-HT concentration in their mPFC and led to enhancement of freezing behavior (44). They proposed that there is a relationship between mPFC levels of 5HT and anxiety state. A relevant study indicated that administration of a selective 5-HT3 agonist, 2-methyl-serotonin, into mPFC could reduce pyramidal neural firing. This effect was reversed by applying a 5-HT3 antagonist (45, 46). Part of the neurons in IL-mPFC innervate the central amygdala (Ce) and stimulate its output projections (47). Output neurons from the Ce are mostly GABAergic. They project to the brainstem and hypothalamic regions and inhibit autonomic or behavioral responses to fearful stimuli (48, 49). The above findings, lead us to speculate that modulation of 5HT3 receptors in mPFC is an important means by which we can manage the fear-related responses. Most cortical 5HT3 receptors are expressed on GABAergic interneurons. These interneurons play important roles in suppressing the activity of the pyramidal projecting neurons (50). Based on these findings, we can propose that ondansetron in our study acts by blockade of the 5HT3 receptors on the above-mentioned interneurons. This probably decreases GABA release and subsequently disinhibits the activity of pyramidal projecting neurons to the Ce (Figure 10). We can propose another mechanism for ondansetron in our study. Several pieces of research have demonstrated that activation of NMDA receptors, placed on the pyramidal neurons, is crucial for the extinction of fear memories (51-56). Evidence from the patch-clamp technique proved that serotonin or its agonist, SR 57227A, inhibited NMDA-induced action potentials in these neurons. It is not fully understood how these ionotropic 5HT3 receptors are coupled to NMDA receptors but it is proposed that perhaps phospholipase-C activating mechanisms are involved (57). Putting these together, we can assume that in our study, ondansetron, as opposed to the previous study, has activated NMDA receptors in IL-mPFC and ultimately increased the output signals to fear-suppressing regions, like the Ce (Figure 10).

The role of the 5-HT3 receptors in extinction consolidation is further supported by the results from our PCR test. Our findings showed that 5HT3A receptors are up-regulated in IL-mPFC of SPS rats (Figure 9). Although there is a huge body of evidence regarding the role of the serotonergic system in the neurobiology of PTSD (58, 59), data on the expression of serotonin receptors is scarce. It was shown, for instance, that overexpression of 5HT1B, as a metabotropic autoreceptor in the dorsal raphe nucleus, will increase anxiety in rats (60). Murrough *et al*. (2011) found that severe trauma exposure reduces 5HT1B receptors in the amygdala, caudate, and the anterior cingulate cortex. Some of the trauma-exposed individuals who have reduced 5HT1B receptors do not eventually develop the PTSD disorder. Therefore, it is argued that the abnormal receptor expression does not necessarily determine the phenotype of PTSD (61). Agonists of the 5HT1A receptor are also found to be effective in the treatment of PTSD. They reduce the release of glutamate in cortical neurons. Reduced glutamate triggers the release of trophic factors in cortical and limbic regions, which induces neurogenesis and impedes neural cell death. These effects have positive outcomes in the treatment of PTSD (59, 62).

As part of the behavioral tests, we have also performed experiments to evaluate the general anxiety of the animals. In the EPM test, consistent with several other studies (63-65), our findings demonstrated that SPS rats have an elevated level of anxiety compared with the normal group (Figure 5). The total number of square crossings did not differ between the SPS and normal rats in the OF test. Therefore, the behavior of the animals in the EPM was not influenced by their locomotor activity (Figure 6). As far as we know, the effect of intra-mPFC administration of a 5HT3 blocker on general anxiety of animals has not been previously evaluated. Our data represents that contrary to its diminishing effects on conditioned fear (Figure 4), repeated microinjections of ondansetron could not alleviate anxiety in SPS rats (Figure 7). Fear and anxiety have many symptoms in common and the neuroanatomical circuits involved partially overlap (66, 67). Therefore, our results imply that 5HT3 receptors in IL-mPFC are probably not an overlapping interface for anxiety and fear.

## Conclusion

Despite extensive knowledge about the neural underpinnings of PTSD, the neurobiological basis of the disease is still poorly understood. In this study, we found that SPS-induced extinction deficits are rescued by blocking of 5-HT3 receptors in IL-mPFC. Meanwhile, SPS rats had enhanced 5HT3 expression in their IL-mPFC region. These results lead us to propose that transcriptional changes of 5HT3 receptors in IL-mPFC are, at least in part, responsible for the impaired extinction of fear in PTSD subjects. It seems that treatment of the disorder with 5HT3 blockers is worth being investigated in clinical practice.
